# Vaccination versus antimicrobials to prevent Porcine Proliferative Enteropathy: associated costs and effects on piglets’ growth, health, and serological performance

**DOI:** 10.3389/fvets.2025.1538206

**Published:** 2025-02-24

**Authors:** Marco Aurélio Gallina, Monike Willemin Quirino, Rafael Frandoloso, Yuso Henrique Tutida, Adriano Norenberg, Arlei Coldebella, Ivan Bianchi, Jalusa Deon Kich

**Affiliations:** ^1^Curso de Pós-Graduação em Produção e Sanidade Animal, Instituto Federal Catarinense, Araquari, SC, Brazil; ^2^MSD Saúde Animal, São Paulo, SP, Brazil; ^3^Laboratório de Microbiologia e Imunologia Avançada, Escola de Ciências Agrárias, Inovação e Negócios, Universidade de Passo Fundo, Passo Fundo, RS, Brazil; ^4^AFK Imunotech, Passo Fundo, RS, Brazil; ^5^Pamplona Alimentos, Rio do Sul, SC, Brazil; ^6^Embrapa Suínos e Aves, Concórdia, SC, Brazil

**Keywords:** pig production, *Lawsonia intracellularis*, vaccination, antibiotics, antimicrobial resistance, antimicrobial prudent use, one health

## Abstract

**Introduction:**

This study evaluated vaccination and prophylactic use of antimicrobials as strategies to prevent Porcine Proliferative Enteropathy (PPE) during nursery and growth-finishing phases.

**Methods:**

Three hundred weaned piglets (~ 29 days old) were distributed into groups: NVMED – no vaccinated against *Lawsonia intracellularis* but in-feed medicated with antimicrobials (amoxicillin, florfenicol, lincomycin, spectinomycin and tilmicosin); VMED – vaccinated and in-feed medicated; VNMED – vaccinated but no in-feed medicated. Piglets were vaccinated at weaning (Porcilis^®^ Ileitis, MSD Animal Health). The following variables were assessed: growth and health performance, anti-*L. intracellularis* IgG levels, *L. intracellularis* fecal shedding, Pneumonia and Pleurisy Index (PPI) at slaughter, antimicrobial consumption and costs, and vaccination expenses.

**Results:**

Average daily gain (ADG) at the nursery phase was lower in VNMED group (*p* < 0.01); however, there was no treatment effect on feed conversion, ADG, and body weight at growth-finishing phase (*p* ≥ 0.23). Similar anti-*L. intracellularis* IgG levels were found for VMED and VNMED groups at all evaluated moments (*p* = 0.01). *L. intracellularis* was only detected in feces samples from 4/90 tested piglets and no difference in health performance was found (*p* > 0.05). Groups presented PPI < 0.89. In-feed antimicrobial consumption and related costs were 3 to 3.5-fold higher for NVMED and VMED groups compared to VNMED group.

**Discussion:**

The prophylactic administration of antimicrobials used in this study did not affect the serological performance post-vaccination against *L. intracellularis*. Additionally, vaccine use to prevent PPE reduced the antimicrobial consumption and related costs by ~70%, with no impairments on production outputs.

## 1 Introduction

Porcine Proliferative Enteropathy is an economically important endemic enteric disease for swine production (1) with a high herd-level prevalence worldwide [varying from 48 to 100%; (2–6)]. The etiological agent, *Lawsonia intracellularis*, can affect piglets older than 4 months, resulting in the acute clinical form (Porcine Hemorrhagic Enteropathy), causing high mortality rates (up to 50%) due to hemorrhagic diarrhea. In contrast, the agent can also infect young piglets (6–20 weeks old) leading to the chronic clinical form (Porcine Intestinal Adenomatosis), which despite resulting in a low mortality rate (~1%) causes significant losses in growth performance due to diarrhea and lesions observed at enterocytes level (6–8). Still, the infection can occur in a silent course (subclinical form), which also causes significant losses in growth performance, being the most common form observed on farms (5).

One of the control approaches for Porcine Proliferative Enteropathy is the use of licensed vaccines (9–16). Currently, three licensed antigen delivery platforms are available to immunize pigs: an attenuated strain of *L. intracellularis* administrated through drinking water or directly into the oral cavity (Enterisol® Ileitis; Boehringer Ingelheim) and two inactivated vaccines administered either intramuscularly (Porcilis® Ileitis & Porcilis® Lawsonia; MSD Animal Health) or intradermally (Porcilis® Lawsonia ID; MSD Animal Health). Porcine Proliferative Enteropathy can also be controlled through the prophylactic use of antimicrobials, which are included in the feed supplied to animals (17, 18), and nowadays the association of vaccines against *L. intracellularis* and prophylactic antimicrobial treatment is widely applied in swine herds (9–16).

In this scenario it is important to understand better how the interaction between antimicrobials and vaccines occurs since, beyond their antibiotic effects, antimicrobials may also modulate the immune response, as already shown for chickens and pigs (19–22). The therapeutic use of antimicrobials in pigs concurrently with the vaccination protocol against viral diseases, such as influenza and Aujeszky, either suppressed the post-vaccinal humoral immune response (21) or negatively affected the cell-mediated response (20). It was also shown that vaccination of pigs against erysipelas during the therapeutic use of antimicrobials decreased or enhanced the production of specific antibodies, according to the antibiotic type (23).

Regardless of the etiological agent, data concerning the possible interactions between the immune system post-vaccination and the prophylactic antimicrobial therapy in swine production are scarce. In respect to *L. intracellularis*, the few information available showed that Anti*-L. intracellularis* IgG levels 5 days after vaccination were lower in piglets medicated with three different antimicrobials than in piglets only vaccinated (24). Nonetheless, no data concerning the animals’ clinical and zootechnical performance was reported. In this sense, several studies reported that piglets vaccinated against *L. intracellularis* and submitted to prophylactic antimicrobial therapy over nursery and growth-finishing phases have shown greater performance compared to pigs that only received the prophylactic therapy (9–15, 25). Nevertheless, these investigations neither assessed piglets’ immune response nor considered a group of piglets vaccinated but not submitted to prophylactic antimicrobial therapy.

It is also worth noting that the ‘One Health’ concept raised important concerns regarding the antimicrobials’ prolonged usage in animal production, especially as prophylactic strategy, due to its contribution to the rise of antimicrobial resistance (26, 27). Around 73% of all antimicrobials sold globally have been used in food-producing animals (28) and the global antimicrobial use for cattle, sheep, chicken, and pigs in 2020 was estimated at 99,502 tones of active ingredients (29). In this sense, the animal food-producing sector, including the swine industry, is facing the challenge of practicing the prudent use of antimicrobials (30), particularly in countries with large herds, such as Brazil. Brazil is the fourth-largest producer and exporter of pork in the world as well as the second-largest consumer of antimicrobials for animal production, presenting increased rates of antimicrobial resistance rates in pig production in the last 20 years (29, 31). This scenario reinforces the necessity and relevance of studies further investigating the possible interactions between immune response after vaccination and prophylactic antimicrobial therapy. Nevertheless, the few studies investigating vaccination to prevent Porcine Proliferative Enteropathy as a strategy to reduce antimicrobial use did not address this topic since their methodology did not consider piglets receiving the vaccine as well as the prophylactic use of antimicrobials or piglets non-vaccinated and receiving the prophylactic use of antimicrobials (16, 32).

Against this background, this study aimed to evaluate the prophylactic use of antimicrobials and vaccination to prevent Porcine Proliferative Enteropathy in a Brazilian swine herd. It compared three groups of piglets: non-vaccinated and medicated, vaccinated and medicated, and vaccinated and non-medicated, evaluating the associated costs and the effects on piglets’ growth, health, and serological performance over the nursery and growth-finishing phases.

## 2 Materials and methods

### 2.1 Ethics statement

The study was approved by the Institutional Committee for Ethical Use of Animals of the Instituto Federal Catarinense – Campus Araquari (protocol no. 396/2022) and followed the Brazilian College of Animal Experimentation guidelines. The experiment was conducted on commercial pig farms located in the southern region of Brazil.

### 2.2 Experimental design

A total of 900 male piglets of the same genetic background from three different commercial sow farms were used in this study. During the suckling period, piglets were vaccinated against *Mycoplasma hyopneumoniae,* porcine circovirus type 2, *Actinobacillus pleuropnemoniae*, *Glaesserella parasuis*, and *Pasteurella multocida D*, as described in Table 1.

**Table 1:** Vaccination protocol applied during the suckling and nursery phases for all piglets used in this study.

Vaccination time	Target agent	Commercial name	Manufacturer	Dosage
7 d post-partum	*Mycoplasma hyopneumoniae*	Respisure One^®^	Zoetis	2 mL (single dose)
21 d and 42 d post-partum	*Actinobacillus pleuropnemoniae. Glaesserella parasuis. Pasteurella multocida* D	Autogenous	Microvet	2 mL (two doses)
Weaning (~ 29 d post-partum)	Porcine circovirus type 2	Circoflex^®^	Böehringer Ingelheim	1 mL (single dose)
Weaning (~ 29 d post-partum)	*Lawsonia intracellularis*	Porcilis^®^ Ileitis	MSD Animal Health	2 mL (single dose)

At weaning, piglets were identified with ear tags, individually weighted and distributed into three groups according to their body weight: NVMED (*n* = 301) – no intramuscular vaccination against *Lawsonia intracellularis* but in-feed medication (antimicrobials with activity against *L. intracellularis* and other agents) over the nursery and growth-finishing phases; VMED (*n* = 297) – intramuscular vaccination against *L. intracellularis* and in-feed medication over the nursery and growth-finishing phases; VNMED (*n* = 302) – intramuscular vaccination against *L. intracellularis* but no in-feed medication over the nursery and growth-finishing phases. The vaccine against *Lawsonia intracellularis* (Porcilis® Ileitis, MSD Animal Health) was administered at weaning (when piglets were ~ 29 d-old) by a single intramuscular injection (2 mL) in the neck region, as recommended by the manufacturer. Stainless steel needles of 20 G × 0.9 mm were used. After weaning, piglets were transferred to a commercial nursery facility (27.27371 S, 49.82817 W) and later moved to a growth-finishing facility (27.14133 S, 49.76669 W).

The nursery and growth-finishing facilities presented the following biosecurity measures: vegetation features, fence around the farm, parking outside the farm, sanitary ford (showers and dressing room), bird-proof nets, and rodent and insect control plans. Furthermore, both farms were positive (molecular detection – qPCR) for *L. intracellularis*.

### 2.3 Management, housing, and feeding – nursery phase

In the nursery phase, piglets were randomly housed in 18 pens (50 piglets/pen; six pens/treatment) for 37 days. During the period, natural ventilation was applied using double curtains on both sides of each nursery room, and each pen was equipped with slatted plastic floors and five nipple drinkers to provide water *ad libitum*. The stocking density was 0.32 m^2^ per animal. Piglets had *ad libitum* access to feed, which was manually offered and the amount loaded was registered. Before housing, the cleaning and disinfection process of the facility was performed using glutaraldehyde- and quaternary ammonium-based disinfectant, followed by a downtime of 5 days.

Animals were fed according to their treatment group and production phase (Table 2). The feed formulation was obtained from the company hosting the study, and the antimicrobial inclusion was performed over the manufacturing process (Table 2). The feed provided to the VNMED group was stored in an exclusive silo. Throughout the study, the use of injectable medications (antibiotic and anti-inflammatory) and number of dead or fallout animals (piglets that were much lighter than their littermates) were recorded. Necropsy was performed on all dead or euthanized piglets during the phase.

**Table 2:** Feeding protocol including nutritional composition and antimicrobial program of diets provided to piglets during the study period.

	Kcal ME/kg	Digestible lysine, %	Phosphorus, %	Antimicrobials	
				NVMED/VMED	VNMED
*Nursery phase*					
Nursery I	3.500	1.50	0.45	Lincomycin + Spectinomycin 44% and Tilmicosin 50%	–
Nursery II	3.500	1.50	0.45		–
Nursery III	3.480	1.50	0.45	–	–
Nursery IV	3.450	1.28	0.45	Tilmicosin 50%	–
Nursery V	3.450	1.28	0.45	–	–
*Growth–finishing phase*					
Growing	3.450	1.10	0.40	Tilmicosin 50% + Amoxicillin 50%	Amoxicillin 50%
Growing I	3.375	1.05	0.35	–	–
Growth support	3.350	1.00	0.35	Florfenicol 30%	Florfenicol 30%
Growing II	3.350	0.95	0.35	–	–
Finishing I	3.360	0.89	0.30	Tilmicosin 50% + Amoxicillin 50%	Amoxicillin 50%
Finishing II	3.380	0.75	0.30	–	–

ME, metabolizable energy; NVMED, Non-vaccinated and medicated group; VMED, Vaccinated and medicated group; VNMED, Vaccinated and non-medicated group.

At the last housing day, piglets were weighted and 868 piglets presenting the highest weight were selected to be housed in a growth-finishing facility.

### 2.4 Management, housing, and feeding – growth and finishing phase

The 868 selected piglets were transferred to a growing and finishing facility and housed in 36 pens. Animals from each nursery pen were divided into two growing and finishing pens; therefore, piglets were housed according to their treatment group (12 pens/treatment) with no mixing of animals from different treatments. The stocking density was 1 m^2^ per pig, and pens had semi-compact concrete flooring along 2/3 of the pen with slatted concrete flooring in the remaining area, equipped with one nipple drinker for every 12 pigs and stainless steel feeder. Before housing, the facility was submitted to the cleaning and disinfection process using glutaraldehyde- and quaternary ammonium-based disinfectants, followed by a downtime period of 5 days.

Diets were also formulated and provided by the company housing the study (Table 1). The food used to feed the VNMED group was stored in an exclusive silo. Pigs were housed for 100 days and during this period the use of injectable medications (antibiotic and anti-inflammatory) and the number of dead or fallout animals were recorded. Necropsy was also performed on all dead or euthanized piglets. Before transporting the animals to the slaughterhouse, they were individually weighted.

### 2.5 Detection of serum anti-*Lawsonia intracellularis* IgG

A total of 30 pigs per treatment was randomly selected and paired blood samples were collected from pigs at different ages (29, 49, 65, 98, 128, and 175 days old). The sample collection was performed by puncture of the jugular vein using polypropylene tubes containing a clotting activator. Blood samples were centrifuged at 500 × *g* for 10 min, and serum samples were collected and stored at −80°C. The detection of anti-*L. intracellularis* IgG was conducted by Flow Cytometry Antibody Test as described by Baldasso et al. (33).

### 2.6 Quantification of *Lawsonia intracellularis* fecal shedding

To evaluate the excretion of *L. intracellularis*, feces samples were collected from the same pigs selected for blood samples collection. Samples were collected directly from the rectal ampoule using a sterile plastic bag and were kept at 2–8°C until arrival at the laboratory. The fecal samples were individually diluted (1 g into 9 mL of PBS pH 7.2) and the total genomic DNA was extracted using the MagMax CORE kit following the manufacturer’s recommendation (ThermoFisher Scientific, USA). The quantitative PCR (qPCR) was conducted according to Stahl et al. (34).

### 2.7 Pneumonia index (PI) assessment

During the slaughter, the severity index of pneumonic lesions was assessed from 150 animals per treatment. Each pulmonary lobe was evaluated and contributed to the scoring based on its proportion related to the total lung area. Lobes were scored according to the extent of pulmonary consolidation lesion: 0%; 0.1–11%; 11.1–21%; 21.1–31%; 31.1–41%; 41.1–51%; 51.1–100% (35). The values of the PPI were classified as pneumonia-free (0.0–0.55); pneumonia with no risk in the herd (0.56–0.89); and high risk of pneumonia in the herd (>0.9).

### 2.8 Economic analysis of vaccination and antimicrobial costs

The total amount of antimicrobials consumed (kg) per group was calculated based on the feed consumption, registered during the nursery and growth-finishing phases, and the amount of antimicrobials (active ingredient) included in the feed. Similarly, the amount of antimicrobials consumed (mg) per kg of live weight in each phase was also estimated, dividing the antimicrobial consumption by the total live weight of the piglets at the end of the nursery and growth-finishing phases. The cost of antimicrobials (per phase; US$) was calculated based on the antimicrobial consumption and the cost of antimicrobials (active ingredient) reported by the feed manufacturing. Additionally, the expenses related to the vaccination against Lawsonia intracellularis were estimated considering only the vaccine cost (number of piglets vaccinated × cost of vaccine’s dose; US$), since there was no additional service cost (Porcilis® ileitis was administered simultaneously with another vaccine – against porcine circovirus type 2 – following the sanitary protocol of the farm.

### 2.9 Statistical analysis

Data were analyzed using the Statistical Analysis System© software (SAS, 2012). The pen was the experimental unit and significant differences were considered when *p* ≤ 0.05. Quantitative responses (body weight – BW, average daily gain – ADG, and feed conversion – FC) were checked regarding residual normality and then analyzed through variance analysis (GLM procedure). Means were compared by the protected t-test. For responses number of dead piglets, number of fallout piglets, and number of piglets treated with injectable drugs a binomial distribution was fitted, and a logistic regression was applied considering the treatment effect (LOGISTIC procedure).

The levels of anti-*L. intracellularis* IgG were analyzed by repeated measures, using the MIXED procedure, selecting the adequate covariance structure based on the lowest value of Akaike Information Criterion (AIC). The treatment, sample moment (piglets’ age), and their interaction were included as fixed effects, comparing the values by the protected t-test.

## 3 Results

### 3.1 Average daily gain, feed conversion ratio, and health performance

The growth performance of piglets from the three groups is presented in Table 3. Feed conversion during the nursery phase was similar amongst the groups (1.46 ± 0.02; *p* = 0.56). Nevertheless, the ADG and the BW at the end of the nursery period were lower for piglets from the VNMED group (350.07 ± 5.79 g, 21.34 ± 0.23 kg; respectively) compared to NVMED (387.46 ± 7.13 g, 22.79 ± 0.26 kg; respectively) and VMED groups (385.04 ± 5.59 g, 22.70 ± 0.21 kg; respectively; *p* < 0.01). Despite that, piglets from VNMED group presented similar BW at the end of the growth and finishing phase compared to piglets from groups NVMED and VMED (131.4 ± 0.69; *p* = 0.75), with no differences in FC or ADG over the growth and finishing phase (*p* = 0.37).

**Table 3:** Zootechnical performance of piglets in the nursery and growth-finishing phases during the study period.

*Nursery phase*	NVMED *n* = 301	VMED *n* = 297	VNMED *n* = 302	*p*-value
Starting weight – kg	8.07 ± 0.02	8.07 ± 0.01	8.03 ± 0.02	0.30
Final weight (37 d) – kg	22.79 ± 0.26^a^	22.70 ± 0.21^a^	21.34 ± 0.23^b^	<0.01
FCR	1.44 ± 0.02	1.46 ± 0.02	1.48 ± 0.03	0.56
ADG (0–37 d) – g	387.46 ± 7.13^a^	385.04 ± 5.59^a^	350.07 ± 5.79^b^	<0.01

*Growth-finishing phase*	NVMED *n* = 287	VMED *n* = 284	VNMED *n* = 297	*p*-value
Starting weight – kg	22.79 ± 0.28^a^	22.73 ± 0.23^a^	21.36 ± 0.25^b^	<0.01
Final weight (110 d) – kg	131.83 ± 0.65	131.24 ± 0.74	131.17 ± 0.67	0.75
FCR	2.33 ± 0.02	2.37 ± 0.02	2.33 ± 0.02	0.23
ADG (0–110 d) – g	991.25 ± 6.36	986.39 ± 6.16	998.36 ± 5.40	0.37

NVMED, Non-vaccinated and medicated group; VMED, Vaccinated and medicated group; VNMED, Vaccinated and non-medicated group; FCR, feed conversion ratio; ADG, average daily gain. Data are presented as means ± standard error of the mean. Significant differences (*p* ≤ 0.05) between groups are indicated by different letters among treatments.

The number of animals treated with injectable medication, the number of fallout piglets, and piglet mortality did not differ amongst groups, regardless of the phase (*p* ≥ 0.10; Table 4).

**Table 4:** Clinical performance of piglets in the nursery and growth-finishing phases during the study period.

*Nursery phase*	NVMED *n* = 301	VMED *n* = 297	VNMED *n* = 302	*p*-value
Treated piglets*, %	6.50 ± 1.06	5.17 ± 0.79	5.50 ± 0.81	0.59
Mortality, %	0.50 ± 0.22	0.83 ± 0.31	0.17 ± 0.17	0.31
Fallout piglets, %**	1.67 ± 0.56	1.33 ± 0.56	0.67 ± 0.49	0.29
Mortality of fallout piglets, %	2.17 ± 0.65	2.17 ± 0.65	0.83 ± 0.65	0.35

*Growth-finishing phase*	NVMED *n* = 287	VMED *n* = 284	VNMED *n* = 297	*p*-value
Treated piglets*, %	0.00 ± 0.00	0.25 ± 0.13	0.25 ± 0.18	1.00
Mortality, %	0.08 ± 0.08	0.67 ± 0.22	0.67 ± 0.22	0.13
Fallout piglets, %**	0.25 ± 0.13	0.42 ± 0.19	0.08 ± 0.08	0.30
Mortality of fallout piglets, %	0.33 ± 0.14	1.08 ± 0.36	0.75 ± 0.22	0.10

*With injectable medication. **Piglets that were much lighter than their littermates. NVMED, Non-vaccinated and medicated group; VMED, Vaccinated and medicated group; VNMED, Vaccinated and non-medicated group. Data are presented as means ± standard error of the mean.

### 3.2 Kinetics of the anti-*Lawsonia intracellularis* antibody response

**Figure 1:**
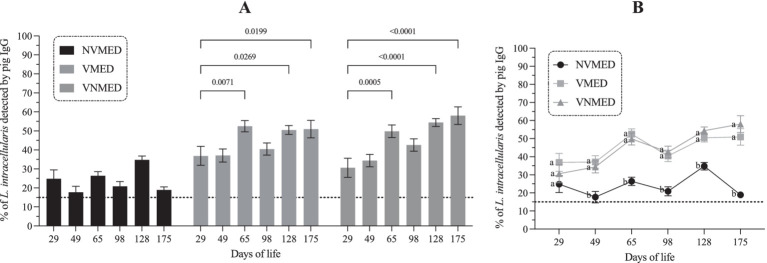
Profile of systemic anti-*Lawsonia intracellularis* IgG in piglets over the study period. **(A)** Antibody response of vaccinated and unvaccinated groups in the presence or absence of antibiotics in the feed. **(B)** Comparison of the antibody curve between the experimental groups. **(A)**: absolute value of “*p*”; **(B)**: different letters indicate significant difference; *p* < 0.05. NVMED, Non-vaccinated and medicated group; VMED, Vaccinated and medicated group; VNMED, Vaccinated and non-medicated group.

As illustrated in Figure 1A, all piglets have detectable levels of maternally derived antibodies at 29 days of age. The levels of systemic anti-*L. intracellularis* IgG observed in vaccinated animals remained stable until day 49 and increased significantly (*p* < 0.05) from day 65 onwards, regardless of the presence or absence of antibiotics in the feed (Figure 1A). On the other hand, levels of anti-*L. intracellularis* IgGs observed in the unvaccinated group decreased from day 29 and at 49 days, only a few animals still had antibodies of maternal origin. Interestingly, we observed two peaks of antibody increase in this group, the first at 65 days of age, and the second at 128 days of age, which indicates that the animals were naturally exposed to *L. intracellularis*. As illustrated in Figure 1B, after application of the vaccine, the antibody curve observed in vaccinated animals compared to unvaccinated animals was completely different. Significant differences (*p* < 0.05) between the vaccinated and non-vaccinated groups were observed from day 49 of life (20 days after vaccine application) until the end of this study.

### 3.3 Shedding profile of *Lawsonia intracellularis* in fecal samples and lung inspection at slaughterhouse

The bacterial agent was only detected in feces samples from four pigs, two from the VMED group (at 49 and 175 days of life) and two from the VNMED group (at 128 and 175 days of life). According to evaluations performed at slaughter, all groups presented animals with pneumonia, showing similar prevalence: 68% (NVMED), 67.3% (VMED), and 70% (VNMED). For all groups, the PPI was <0.89, presenting no risk to the herd (0.73; 0.72; and 0.79, respectively).

### 3.4 Antimicrobial consumption and expenses with antimicrobial use and vaccination

**Figure 2:**
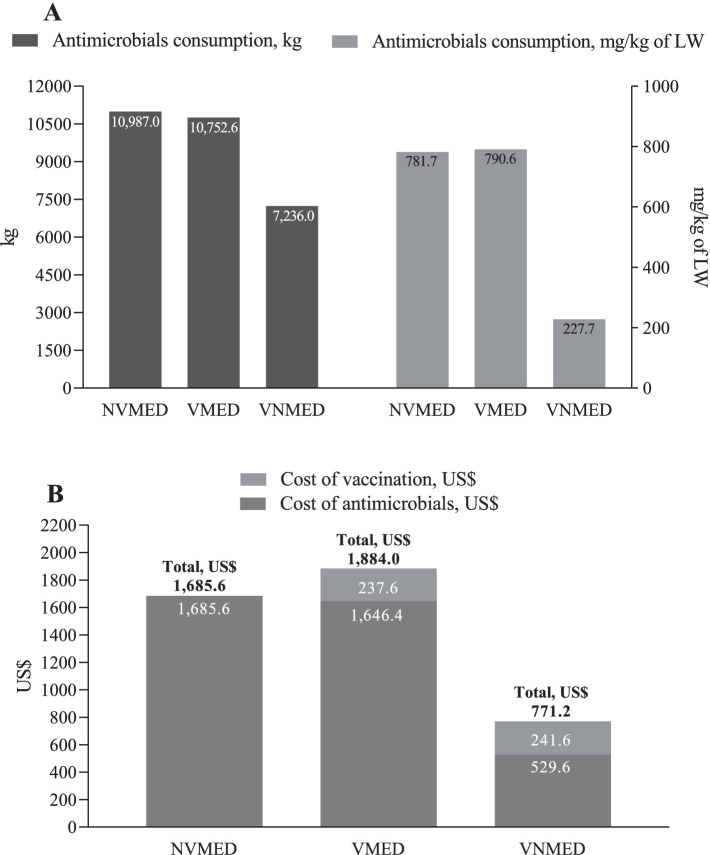
Consumption and costs of in-feed antimicrobials, and cost of vaccination process against *Lawsonia intracellularis* over the study period. **(A)** Total consumption of in-feed antimicrobials over the nursery and growth-finishing phases (kg and mg/kg of live weight). **(B)** Expenses related to the consumption of in-feed antimicrobials and the vaccination process against *Lawsonia intracellularis* during the nursery and growth-finishing phases. NVMED, Non-vaccinated and medicated group; VMED, Vaccinated and medicated group; VNMED, Vaccinated and non-medicated group. LW, live weight. Cost of vaccination service was not considered since Porcilis^®^ ileitis was administered simultaneously to another vaccine, following the sanitary protocol of the farm (against porcine circovirus type 2). Therefore, there was no additional service cost related to vaccination against *Lawsonia intracellularis*.

Antimicrobial consumption and expenses with antimicrobial use and vaccination are presented in Table 5 and Figure 2. Total in-feed antimicrobial consumption (mg/kg of live weight) for NVMED and VMED piglets was ~3.5-fold greater than the consumption for VNMED piglets: 781.7 vs. 790.6 vs. 227.7, respectively; leading to a decrease of 554–563 mg of antimicrobial/kg of live weight (71%). The expenses with antimicrobials throughout the nursery and growth-finishing phases for NVMED and VMED piglets were ~ 3-fold greater than the costs for VNMED piglets: US$ 1,685.62 vs. US$ 1,646.37 vs. US$ 529.56, respectively. These results demonstrate that VNMED group (not treated with antibiotics) generated a cash savings of 68 and 69% compared to the groups that received antibiotic treatment.

**Table 5:** Expenses related to the vaccination process against *Lawsonia intracellularis* and the in-feed antimicrobials consumption during the study period.

*Vaccination expenses*	NVMED *n* = 301	VMED *n* = 297	VNMED *n* = 302
Vaccine cost, US$	0.0	237.6	241.6
Cost of vaccination service^*^, US$	0.0	0.0	0.0
Total cost of vaccination, US$	0.0	237.6	241.6

*Nursery phase*	NVMED *n* = 301	VMED *n* = 297	VNMED *n* = 302
Amount of antimicrobials (kg) per diet			
Nursery I	213.0	213.0	0.0
Nursery II	426.0	421.7	0.0
Nursery III	660.0	651.2	0.0
Nursery IV	798.8	798.0	0.0
Nursery V	0.0	0.0	0.0
Antimicrobials consumption, kg	2.097.8	2.083.9	0.0
Antimicrobials consumption, mg/kg of live weight	494.4	500.5	0.0
Cost of antimicrobials, US$	153.1	152.2	0.0

*Growth-finishing phase*	NVMED *n* = 287	VMED *n* = 284	VNMED *n* = 297
Amount of antimicrobials (kg) per diet			
Growing	3,440.0	3,422.8	1,694.2
Growing I	0.0	0.0	0.0
Growth support	1,393.2	1,345.9	1,401.8
Growing II	0.0	0.0	0.0
Finishing I	4,056.0	3,900.0	4,140.0
Finishing II	0.0	0.0	0.0
Antimicrobials consumption, kg	8,889.2	8,668.7	7,236.0
Antimicrobials consumption, mg/kg of live weight	287.2	290.1	227.7
Cost of antimicrobials, US$	1,532.5	1,494.2	529.6

NVMED, Non-vaccinated and medicated group; VMED, Vaccinated and medicated group; VNMED, Vaccinated and non-medicated group. *Cost of vaccination service was not considered since Porcilis^®^ ileitis was administered simultaneously with another vaccine, following the sanitary protocol of the farm (against porcine circovirus type 2). Therefore, there was no additional service cost related to vaccination against *Lawsonia intracellularis*.

## 4 Discussion

*Lawsonia intracellularis* is a highly prevalent microorganism in Brazil (36) that causes porcine proliferative enteropathy. The diagnosis of this disease can be easily performed when the animals present typical clinical signs of the acute and chronic forms of the disease. On the other hand, when the infection presents subclinically, which occurs most of the time, the microorganism is only detected through laboratory diagnosis. In this study, we conducted a field experiment to evaluate the clinical, zootechnical performance and immune profile of a group of piglets vaccinated against *L. intracellularis* (VNMED) compared to a group of piglets vaccinated and medicated with antibiotics (VMED) and/or non-vaccinated and medicated with antibiotics (NVMED).

As expected, piglets vaccinated at 29 days of age with the Porcilis® Ileitis vaccine developed a clear systemic IgG response after vaccination (Figure 1). This vaccine is formulated with an inactivated strain of *L. intracellularis* and potentiated with an oil-based adjuvant (37). The immunogenicity of this vaccine has already been demonstrated in controlled immunization and experimental challenge studies (37), and here, in a field experiment. In our study, piglets that received the vaccination showed significantly more antibodies than those not immunized. Furthermore, using the Flow Cytometry Antibody Test (33), we observed that lincomycin, spectinomycin, tilmicosin, and amoxicillin did not reduce the levels of specific IgG; therefore, they can be used concomitantly to the genesis of the adaptive response. On the other hand, ceftiofur, doxycycline, and tulathromycin may significantly reduce the antibody response if used during the development of the vaccine response (24).

To control *L. intracellularis* infection, pigs can use humoral (antibodies) and cellular (T helper 1 and cytotoxic T lymphocytes) immune responses. These responses can be modulated during the natural infection process (38) or by vaccination (39). Regarding the antibody response, we observed that vaccinated pigs presented high levels of IgG for more than 20 weeks, confirming the duration of immunity of this vaccine (40). As described in Figure 1A, few animals presented positive levels of IgG at 49 days of age, demonstrating that the duration of maternal immunity was ≤7 weeks in this study. An increase in antibody levels was observed at 65 days of age, demonstrating the occurrence of natural infection throughout the nursery phase. Interestingly, at this moment, it was not possible to detect the excretion of *L. intracellularis* in feces.

In a recent study conducted in Brazil, it was reported that the peak of *L. intracellularis* fecal shedding occurs when piglets are 90–120 days old (36). Despite that, in this study, none of the piglets from group NVMED presented fecal samples positive for *L. intracellularis*. Nevertheless, this low shedding may be related to a low infection pressure, which in this study was a consequence of the vaccination or medication process, since all piglets from VNMED, VMED, and NVMED groups were housed in the same farm and facility. Unfortunately, a group of piglets non-vaccinated and non-medicated was not considered in our methodology, which is a limitation that also contributed to a low agent shedding. Despite that, we must highlight that although this trial was performed in a farm positive for *L. intracellularis* a low shedding was observed even for the piglets vaccinated but not medicated, showing that vaccination was successfully effective in controlling the agent, regardless of the antimicrobial use. Furthermore, the prophylactic use of antimicrobials was not crucial to avoid respiratory diseases, given that the PPI did not differ amongst the three groups.

Concerning the piglets’ performance, the few studies investigating the effects of vaccination against *L. intracellularis* on piglets’ growth performance within a scenario of no in-feed antimicrobial use reported a greater average daily weight gain when vaccination was implemented (1, 32). Contrarily, in our study, piglets vaccinated and not medicated (VNMED) had lower average daily weight gain and, consequently, lower final weight at the nursery phase compared to piglets non-vaccinated and medicated (NVMED) and piglets vaccinated and medicated (VMED). Nevertheless, at the growth-finishing phase, the VNMED piglets overcame the lower starting weight – probably due to a compensatory effect – reaching similar average daily weight gain and body weight to other piglets. Regardless, it is important to highlight that savings obtained through the VNMED scenario would probably overcome the expenses related to the lower final weight in VNMED piglets at the nursery phase. Our results showed that the reduction of ~71% in antimicrobials consumption in the VNMED scenario led to an economy of US$ 1,156.0 or 1,116.8 compared to the NVMED or VMED approach, respectively (savings of 68 and 69%, respectively). Even when vaccination expenses were considered, an economy of US$ 914.4 (46% of the total costs for NVMED) or US$ 1,112.8 (59% of the total costs for VMED) was reached. This significant cost saving are attributed to the absence of in-feed antimicrobial use during the nursery phase and the low cost of antimicrobials used during the growth-finishing phase for the VNMED group. Although the VNMED group consumed 7,236.0 kg of antimicrobials in the growth and finishing phase – representing 81 to 83% of the consumption of the NVMED and VMED groups (8,889.2 kg and 8,668.7 kg, respectively) – the majority of this consumption was of a lower-cost antimicrobial (amoxicillin) compared to the one most used in the feed for NVMED and VMED groups (tilmicosin).

The overall results of our study, especially the reduction of ~71% in antimicrobial consumption in the VNMED group, comply with the ‘One Health’ premise of reducing antimicrobial use in animal production and, consequently, antimicrobial resistance, which is one of the global challenges that intensive animal production needs to collaborate on in its confrontation. The swine production presented the largest projected increase in antimicrobial consumption and contributed 45% to the total increase between 2017 and 2030. In 2020, the global antimicrobial use intensity in pigs was estimated at 173.1 mg/PCU (population correction units), while for cattle and chicken, it was 59.6 mg/PCU and 35.4 mg/PCU, respectively (28, 29, 41). Mitigating antimicrobial resistance in animals, humans, and the environment depends primarily on reducing the need for antimicrobial use. Nevertheless, currently, the antimicrobial use in pig production seems to be directly related to the common fear among farmers and veterinarians, who believe that a scenario of reduced use of antimicrobials would not result in lower production outputs; therefore, the high use of antimicrobials would still be necessary to support intensive production (42). Thus, the findings of our study are also essential for decision-makers in the swine industry.

The lack of a group of piglets non-vaccinated and non-medicated is an important limitation of our study. Considering non-vaccinated and non-medicated piglets – besides the non-vaccinated and medicated, vaccinated and medicated, and vaccinated and non-medicated groups – to further evaluate the vaccination as a strategy to reduce the use of antimicrobials in swine production is one of the future perspectives for this topic, as well as considering a different sanitary challenge and comparing the vaccination with other alternative approaches [i.e., use of additives; (43)]. Nevertheless, the overall data found in this study strongly suggest that prophylactic use of antimicrobials in swine production may not be needed to achieve satisfactory health and growth performance when vaccination protocols and adequate biosecurity measures are appropriately applied (44), besides leading to an expressive decrease in antimicrobials use and expenses.

## 5 Conclusion

The use of inactivated vaccine against *L. intracellularis* to prevent Porcine Proliferative Enteropathy is an effective strategy to reduce the prophylactic use of antimicrobials. Replacing the drug program with vaccination did not change the zootechnical parameters; however, it significantly reduced the expenses with antimicrobials, increasing the profitability of the operation.

## Data Availability

The raw data supporting the conclusions of this article will be made available by the authors, without undue reservation.
